# Diagnostic Value of Perfusion Parameters for Differentiation of Underlying Etiology in Internal Carotid Artery Occlusions

**DOI:** 10.1007/s00062-023-01349-0

**Published:** 2023-10-26

**Authors:** Daniel Weiss, Henrik Lang, Christian Rubbert, Kai Jannusch, Marius Kaschner, Vivien Lorena Ivan, Julian Caspers, Bernd Turowski, Robin Jansen, John-Ih Lee, Tobias Ruck, Sven Günther Meuth, Michael Gliem

**Affiliations:** 1 Department of Diagnostic and Interventional Radiology, Medical Faculty, University Düsseldorf, Moorenstraße 5, 40225 Düsseldorf, Germany; 2 Department of Neurology, Medical Faculty, University Düsseldorf, Moorenstraße 5, 40225 Düsseldorf, Germany

**Keywords:** Stroke, Dissection, Embolic, Macroangiopathy, Endovascular treatment

## Abstract

**Purpose:**

Occlusions of the internal carotid artery (ICA) may be caused by dissection, embolic or macroangiopathic pathogenesis, which partially influences the treatment; however, inferring the underlying etiology in computed tomography angiography can be challenging. In this study, we investigated whether computed tomography perfusion (CT-P) parameters could be used to distinguish between etiologies.

**Methods:**

Patients who received CT‑P in acute ischemic stroke due to ICA occlusion between 2012 and 2019 were retrospectively analyzed. Group comparisons between etiologies regarding the ratios of CT‑P parameters between both hemispheres for relative cerebral blood volume (rCBV), relative cerebral blood flow (rCBF), time to maximum (Tmax), and mean transit time (MTT) were calculated by one-factorial analysis of variance (ANOVA) and compared by pairwise Bonferroni post hoc tests. An receiver operating characteristics (ROC) analysis was performed if differences in group comparisons were found. Multinomial logistic regression (MLR) including pretherapeutic parameters was calculated for etiologies.

**Results:**

In this study 69 patients (age = 70 ± 14 years, dissection = 10, 14.5%, embolic = 19, 27.5% and macroangiopathic = 40, 58.0%) were included. Group differences in ANOVA were only found for MTT ratio (*p* = 0.003, η^2^ = 0.164). In the post hoc test, MTT ratio showed a differentiability between embolic and macroangiopathic occlusions (*p* = 0.002). ROC analysis for differentiating embolic and macroangiopathic ICA occlusions based on MTT ratio showed an AUC of 0.77 (*p* < 0.001, CI = 0.65–0.89) and a cut-off was yielded at a value of 1.15 for the MTT ratio (sensitivity 73%, specificity 68%). The MLR showed an overall good model performance.

**Conclusion:**

It was possible to differentiate between patients with embolic and macroangiopathic ICA occlusions based on MTT ratios and to define a corresponding cut-off. Differentiation from patients with dissection versus the other etiologies was not possible by CT‑P parameters in our sample.

## Introduction

Occlusions of internal carotid artery (ICA) may cause cerebral ischemia directly but also display risk for delayed ischemic stroke [1, 2]. The occlusion itself may be caused by different etiologies: by progressive macroangiopathic changes or a sudden occlusion occurs due to embolic thrombus or a dissection [3].

A dissection is usually caused by an intimal tear within the wall of the ICA and a resulting detachment of the intima due to the blood flow along the media, often due to minor trauma of the neck or in the context of vasculopathy [4]. Macroangiopathy is defined as a slowly progressing stenosis with eventual occlusion of the ICA by atherosclerotic processes, whereas an embolic occlusion is caused by a sudden thromboembolic event [5].

Depending on the underlying etiology of the ICA occlusion, treatment strategies are developed, and further care is planned [6, 7]. For example, stent implantation may be necessary in macroangiopathic but not in embolic occlusions. Therefore, timely knowledge of the underlying etiology is helpful to optimize treatment regimens of affected patients.

However, determination of the etiology of ICA occlusion may be difficult [8, 9]. Indications for the underlying etiology may be derived from demographic variables such as patient age, i.e., dissections are more common in young patients, whereas macroangiopathic occlusions are most frequently found in older populations [10]. On the one hand, infarct patterns or typical presentation in stroke imaging, i.e., computed tomography (CT) and computed tomography angiography (CT-A), may give gross information. Recently, CT thrombus characteristics, such as attenuation and length have been identified to contribute to the etiologic identification [11].

Another approach to analyze cerebrovascular reactivity non-invasively is offered by cerebral perfusion (CT-P) [12]. Indeed, the etiologies evaluated are distinguishable on the basis of their collateral status: e.g., macroangiopathic occlusions are often better collateralized than embolic occlusions, because the development of occlusion is much longer in the former and, thus, a more appropriate collateral supply can be formed [13]. Due to the different collateralization of the individual etiologies, the differentiation of these etiologies based on CT‑P is reasonable; however, currently there are no specific investigations on the prediction of the etiology by CT‑P parameters.

Therefore, the aim of this study was to investigate whether differentiation between the etiologies of ICAocclusion is possible based on cerebral perfusion parameters on CT‑P imaging.

## Material and Methods

### Patient Selection

The retrospective study was approved by the local institutional ethics board (ID: 4743R). The requirement for written informed consent was waived. All patients who had suffered an acute ischemic stroke caused by an occlusion of the ICA at the local radiology department between 2012 and 2019 were retrospectively enrolled.

**Fig. 1:**
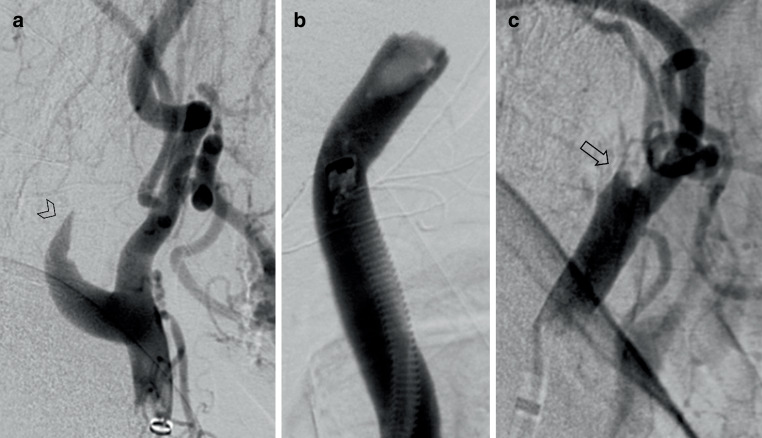
Angiographic images of different etiologies of internal carotid artery (ICA) occlusions. Angiographic images in lateral projection of (**a**) a dissection of the ICA with vessel wall irregularities and string sign (*arrowhead*), **b** a thromboembolic occlusion of the ICA and distal common carotid artery with a sudden, irregularly bordered filling defect, **c** a macroangiopathic occlusion of the ICA with an hourglass-shaped, irregularly bordered tapering of the vessel and sudden filling defect (*open arrow*); Contrast agent was administered via 8F arrow sheath in these examinations

Patients were included if they met the following criteria: (1) occlusion of the ICA, (2) no high-grade contralateral ICA stenosis, (3) sufficient pretreatment computed tomography perfusion (e.g., good opacification, low movement artifacts) and patent distal cerebrovascular arteries as detected by CTA (especially no tandem-occlusions) and (4) available information on the underlying etiology. A structured, interdisciplinary procedure was employed to determine the etiology of carotid occlusion in accordance with current guidelines. Clinical information, including neurological symptoms and medical history, was supplemented by findings from imaging modalities such as CT-A, digital subtraction angiography (DSA), and color-coded duplex sonography. A cardiologic assessment was conducted to investigate any potential sources of thrombus, incorporating echocardiography, duplex ultrasounds, 24‑h Holter electrocardiography, and relevant laboratory diagnostics. The diagnosis of the precise etiology of the stroke was established through a combination of these clinical tests and imaging observations, such as those obtained during endovascular treatment (e.g., occluded macroangiopathic stenosis, visible dissection, or thrombus occlusion without underlying carotid pathology). Additionally, the Trial of Org 10172 in Acute Stroke Treatment (TOAST) criteria were applied in the diagnostic process [14]: (1) for a macroangiopathic occlusion, calcifications of the carotid artery must be present, which can lead to typical changes, e.g., by Doppler and color-coded duplex ultrasound, and at the same time, neurological symptoms must not be reasonably explained by another cause; (2) for an embolic occlusion, a confirmed source of embolism must be present, without, e.g., extensive arteriosclerosis; (3) for a dissection, a source of embolism and extensive arteriosclerosis must be excluded and typical morphological criteria, e.g., in DSA, magnetic resonance imaging or CT‑A (i.e., string sign or intramural hematoma), must be fulfilled [10]. Typical occlusion patterns may be seen in CT‑A or DSA with higher sensitivity of the latter (Fig. 1; [15]). Only patients in whom the etiology could be determined with reasonable certainty were included. Patients included are those who received intravenous thrombolysis (IVT), endovascular thrombectomy (ET), a combination of both therapeutic options and no therapy (Table 1).

**Table 1:** Baseline data

	Total	Dissection	Embolic	Macroangiopathic	*p*-value
Sample size, *n*	69	10	19	40	–
Age, mean, SD (min-max) in years	70 (±14, 28–102)	54 (±11, 28–66)	81 (±11, 57–102)	69 (±12, 48–93)	*<* *0.001*
Female sex, *n* (%)	26 (37.7)	1 (10.0)	11 (57.9)	26 (65.0)	*0.037*
Left hemisphere, *n* (%)	35 (50.7)	8 (80.0)	9 (47.4)	18 (45.0)	0.137
*History*					
Hypertension, *n* (%)	56 (81.2)	5 (50.0)	15 (78.9)	36 (90.0)	*0.016*
Diabetes mellitus, *n* (%)	15 (21.7)	0 (0.0)	7 (36.8)	8 (20.0)	0.070
Atrial fibrillation, *n* (%)	21 (30.4)	0 (0.0)	18 (94.7)	3 (7.5)	*<* *0.001*
Previous stroke, *n* (%)	7 (10.1)	1 (10.0)	2 (10.5)	4 (10.0)	0.998
NIHSS at admission, median (IQR)	5 (3–11)	7 (4–14)	6 (3–12)	4 (2–10)	0.272
mRS at admission, median (IQR)	4 (2–5)	4 (3–4)	5 (3–5)	3 (2–5)	0.069
*CT perfusion parameters*					
rCBF ratio, mean (SD, min-max)	0.82 (0.21, 0.40–1.68)	0.73 (0.16, 0.57–0.95)	0.91 (0.30, 0.42–1.68)	0.81 (0.15, 0.40–1.13)	0.060
rCBV ratio, mean (SD, min-max)	1.01 (0.28, 0.42–2.34)	0.95 (0.22, 0.49–1.22)	1.02 (0.42, 0.42–2.34)	1.02 (0.20, 0.59–1.41)	0.776
MTT ratio, mean (SD, min-max)	1.21 (0.24, 0.75–1.76)	1.24 (0.26, 0.75–1.76)	1.06 (0.15, 0.81–1.31)	1.28 (0.24, 0.81–1.76)	*0.003*
Tmax ratio, mean (SD, min-max)	2.09 (0.95, 0.49–4.85)	2.47 (0.96, 1.44–4.10)	1.78 (0.88, 0.49–3.88)	2.14 (0.95, 0.97–4.85)	0.155
*Acute treatment*					
None, *n* (%)	40 (58.0)	4 (40.0)	13 (68.7)	23 (57.5)	0.341
IVT alone, *n* (%)	9 (13.0)	1 (10.0)	2 (10.5)	6 (15.0)	0.853
ET alone, *n* (%)	7 (10.1)	0 (0.0)	2 (10.5)	5 (12.5)	0.508
IVT and ET, *n* (%)	13 (18.8)	5 (50.0)	2 (10.5)	6 (15.0)	*0.024*
*Procedural data (n* *=* *20)*					
Door-to-IVT, mean (SD, min-max) in min	59 (±48, 10–155)	48 (±22, 28–71)	95 (±94, 28–230)	49 (±23, 18–92)	0.257
Onset-to-IVT, mean (SD, min-max) in min	108 (±48, 37–224)	94 (±17, 75–108)	120 (±90, 64–224)	108 (±44, 37–211)	0.827
Door-to-groin puncture, mean (SD, min-max) in min	90 (±32, 30–150)	100 (±21, 86–136)	58 (±29, 30–88)	97 (±31, 39–150)	0.067
Onset-to-groin puncture, mean (SD, min-max) in min	220 (±147, 39–541)	341 (±200, 121–541)	212 (±121, 108–378)	156 (±83, 39–325)	0.070
TICI (2b–3), *n* (%)	15 (75.0)	4 (80.0)	3 (75.0)	8 (72.7)	0.955
*Clinical outcome at discharge*					
NIHSS, median (IQR)	2 (0–10)	4 (0–11)	5 (2–10)	2 (0–10)	0.427
NIHSS shift, median (IQR)	2 (0–4)	4 (2–4)	1 (0–4)	2 (0–3)	0.261
mRS, median (IQR)	2 (1–4)	2 (1–3)	4 (2–4)	2 (0–4)	0.114

*p*-value refers to an ANOVA for interval-scaled variables and to a Kruskal-Wallis-test for categorial variables

*NIHSS* National Institutes of Health Stroke Scale, *mRS* modified Rankin Scale, *CT* computed tomography, *rCBF* relative cerebral blood flow, *rCBV* relative cerebral blood volume, *MTT* mean transit time, *Tmax* time to maximum, *IVT* intravenous thrombolysis, *ET* endovascular treatment, *TICI* thrombolysis in cerebral infarction, *IQR* interquartile range

### Treatment

The indications for IVT were determined by the neurologist and for ET by the neuroradiologist and neurologist on duty in accordance with current guidelines. The procedure of ET was performed depending on the underlying etiology. Therapeutic concepts differed according to this and may include, for example, thrombectomy or implantation of a stent.

### Imaging and Image Analysis

Patients were examined with a standardized stroke protocol including non-contrast enhanced cranial computed tomography (CT), CT angiography (CT-A) and CT‑P.

The CT‑P was acquired with two adjacent slices of 1 cm thickness angled parallel to the Frankfurt horizontal line at the level of the cella media over 50 s with one image per second. The CT‑P parameters were 80 kVp, 180 mA, 1 × 10 mm collimation (SOMATOM Definition FLASH, Siemens Healthcare GmbH, Erlangen, Germany) or 80 kVp, 120 mA, 64 × 0.6 mm collimation (SOMATOM Definition AS, Siemens Healthcare GmbH, Erlangen, Germany). For CT‑P, a second bolus of contrast agent of 30 ml iomeprol 400 followed by a 30 ml saline solution bolus was injected at 5 ml/s. Image acquisition started with a 3s delay after injection.

Perfusion maps including time to maximum (Tmax), mean transit time (MTT), relative cerebral blood volume (rCBV), and relative cerebral blood flow (rCBF) were calculated using singular value decomposition (STROKETOOL-CT, Version 2.0, H.-J. Wittsack, Digital Image Solutions, Frechen, Germany). The arterial input function was determined automatically or, if automatic detection failed, manually by selecting up to 10 reference voxels in the most opacified arterial vessels. Subsequent extraction of territorial values of the MCA territory from CT‑P parameter maps was done using Angiotux CT 2D (ECCET 2006, Dr. Andreas Beck, Langenfeld, Germany). Ratios of perfusion parameters were used to adjust for interindividual differences in cerebral perfusion. For this purpose, mean values of affected MCA territory were divided by the mean values of the unaffected MCA territory to calculate ratios of the perfusion parameters.

### Outcome Analysis

Functional outcome of patients was evaluated using the modified Rankin Scale (mRS) at discharge. Assessment of mRS was obtained at discharge by medical reports and mRS ≤ 2 was considered as favorable outcome and mRS ≥ 3 as poor outcome.

### Statistical Analysis

Statistical analysis was performed with SPSS software environment (Statistical Package for Social Science, version 28, IBM, Armonk, NY, USA). A *p* value < 0.05 was considered statistically significant for all analyses.

One way ANOVA with etiologies (macroangiopathic, embolic, dissection) as independent variable and ratio of the respective perfusion parameter as dependent variable was calculated for each perfusion parameter (rCBF, rCBV, MTT, Tmax). Furthermore, one way ANOVA with etiologies as independent variable and age as dependent variable was calculated. Bonferroni post hoc analysis was conducted if statistically significant results were reported by the ANOVA. Effect size was calculated using η^2^. For categorical variables, i.e., baseline characteristics except for age, Kruskal-Wallis test with Bonferroni post hoc analyses were calculated with etiologies as independent variable and the categorial baseline characteristic as dependent variable (Table 1).

For parameters, in which differentiation between two etiologies based on a perfusion parameter was possible, a subsequent ROC analysis was performed and the area under the curve (AUC) as well as confidence intervals (CI) were calculated. Furthermore, optimal Youden’s index was calculated to establish a cut off value.

Multinomial logistic regression was calculated for etiologies (macroangiopathic, embolic, dissection) as dependent variables. The model included pretherapeutic parameters, i.e., age, sex, hypertension, atrial fibrillation and MTT ratio as independent variables. Likelihood ratio, goodness of fit and pseudo-Nagelkerkes R^2^ were calculated.

## Results

A total of 69 patients met the inclusion criteria for the current analysis. Mean age was 70 (±14) years and 37.7% of all patients were female. Median NIHSS at admission was 5 (IQR 3–11) and median mRS at admission was 4 (IQR 2–5). Of the patients 40 (58.0%) did neither receive an IVT or an ET, 9 (13.0%) were undergoing IVT alone, 7 (10.1%) were undergoing ET alone and 13 (18.8%) were undergoing both. Of those patients who were undergoing ET, 15 (75.0%) achieved a favorable recanalization (TICI 2b–3). Clinical outcome was determined at discharge: median NIHSS was 2 (IQR 0–10) and median mRS was 2 (0–4). Tendentially worst outcome was achieved in patients with an embolic ICA occlusion with a median of 4 (IQR 0–4) (Table 1).

Age (*p* < 0.001) did differ between the three different etiologies in one-way ANOVA with a strong effect size (η^2^ = 0.54). In a post hoc Bonferroni test between pairs of etiologies, we could show differences between every pair of etiologies, namely between embolic occlusions (mean age 81 ± 11 years) and dissections (mean age 54 ± 11 years) (*p* < 0.001), dissections and macroangiopathic occlusions (mean age 69 ± 12 years) (*p* = 0.002) and macroangiopathic and embolic occlusions (*p* < 0.001) (Table 2). Presence of atrial fibrillation differed between etiologies in Kruskal-Wallis test (*p* < 0.001) and occurred more frequently in patients with embolic occlusions compared to dissections (*p* < 0.001) and macroangiopathic occlusions (*p* < 0.001). Presence of hypertension also differed between etiologies in Kruskal-Wallis test (*p* = 0.016) and occurred more frequently in patients with macroangiopathic occlusions than dissections (*p* = 0.012). Sex differed between etiologies in Kruskal-Wallis test (*p* = 0.037) whereas males were more frequently affected by dissections compared to embolic occlusions (*p* = 0.042). Combined use of IVT and ET differed between etiologies in Kruskal-Wallis test (*p* = 0.024) but did not show distinct differences in pairwise post hoc comparisons. All further baseline characteristic did not show significant differences between the three etiologies.

**Table 2:** Analysis of variance (ANOVA) with post hoc Bonferroni test for age

Etiologies	Post hoc Bonferroni test
Dissection/embolic	p < 0.001, 26.33, 95% CI 15.34–37.33
Dissection/macroangiopathic	p = 0.002, 14.50, 95% CI 4.55–24.45
Embolic/macroangiopathic	p < 0.001, 11.83, 95% CI 3.99–19.67

One-way ANOVA showed substantial group differences for age (F (2.66) = 17.79, *p* < 0.001, η^2^ = 0.350) with a remarkable effect size. A post hoc Bonferroni test was conducted and showed substantial differences between all etiologies

*CI* confidence interval

**Fig. 2:**
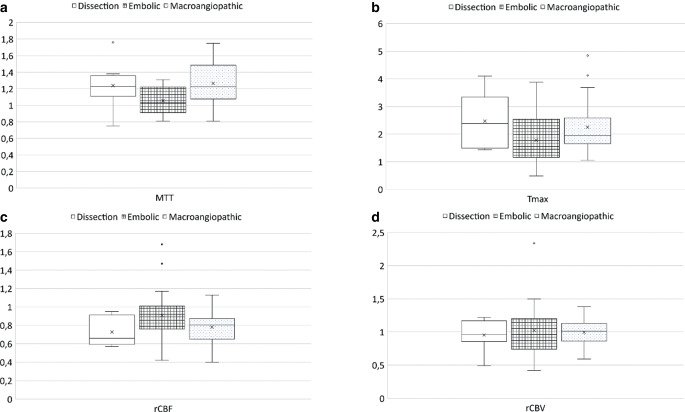
*Boxplots* of perfusion parameters of patients with a dissection, an embolic and a macroangiopathic occlusion of the internal carotid artery (ICA) for MTT (**a**), Tmax (**b**), rCBF (**c**) and rCBV (**d**). *rCBV* relative cerebral blood volume, *rCBF* relative cerebral blood flow, *MTT* mean transit time, *Tmax* time to maximum, *MCA* middle cerebral artery; visualization of perfusion parameters in different etiologies: ratios were formed by dividing mean values of affected MCA territory by the mean values of the unaffected MCA territory

Regarding CT‑P analysis, rCBF ratio (*p* = 0.060), rCBV ratio (*p* = 0.776) and Tmax ratio (*p* = 0.155) did not differ in one-way ANOVA between etiologies (Table 3); however, there was a significant difference between etiologies for MTT ratio (*p* = 0.003) with a strong effect size (η^2^ = 0.164). The post hoc Bonferroni test showed differences in MTT ratio between embolic and macroangiopathic ICA occlusions (*p* = 0.002) but not between embolic occlusions and dissections or between dissections and macroangiopathic occlusions (Table 4). A visualization of perfusion parameter ratios is shown in Fig. 2.

**Table 3:** Analysis of variance (ANOVA) with Computed tomography perfusion parameters and etiologies

CT perfusion parameter	ANOVA
rCBF ratio	F (2.66) = 2.9, p = 0.060; η^2^ = 0.081
rCBV ratio	F (2.66) = 0.3, p = 0.776; η^2^ = 0.008
MTT ratio	F (2.66) = 6.5, p = 0.003; η^2^ = 0.164
Tmax ratio	F (2.66) = 1.9, p = 0.155; η^2^ = 0.055

One-way ANOVA showed substantial group differences solely for MTT ratio with a remarkable effect size. A post hoc Bonferroni test was conducted for MTT

*CT* computed tomography, *rCBV* relative cerebral blood volume, *MTT* mean-transit-time; *Tmax* time to maximum, *IVT* intravenous thrombolysis; *η*^*2*^*(eta-squared)* effect size

**Table 4:** Post hoc Bonferroni test for mean transit time (MTT)

Etiologies	Post hoc Bonferroni test
Dissection/embolic	p = 0.125, 0.18, 95%-CI [−0.03–0.39]
Dissection/macroangiopathic	p = 1.000, −0.04, 95%-CI [−0.23–0.15]
Embolic/macroangiopathic	p = 0.002, 0.22, 95%-CI [0.07–0.37]

Post hoc Bonferroni test showed solely a substantial difference between embolic and macroangiopathic occlusions for MTT

*MTT* meant transit time

**Fig. 3:**
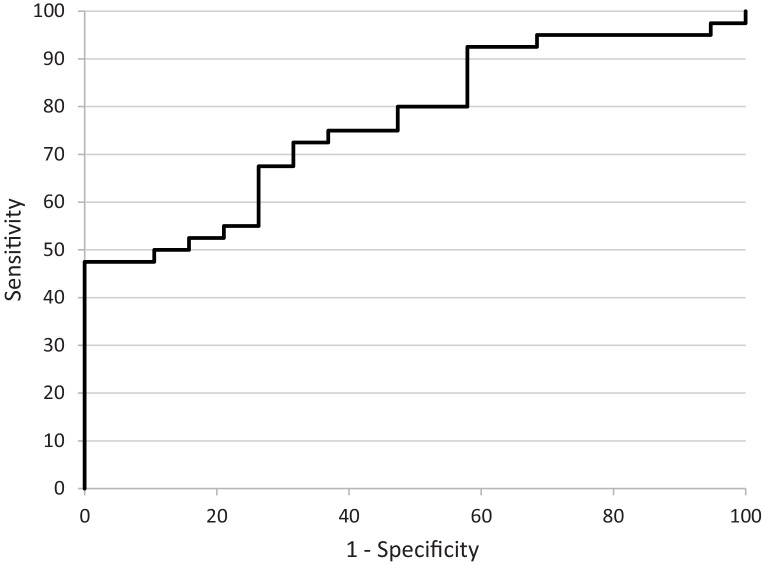
Receiver operating characteristic analysis. Receiver operating characteristic curves for mean transit time (*MTT*) ratio to distinguish between embolic or macroangiopathic occlusions of the internal carotid artery

Subsequent ROC analysis for MTT ratio between embolic and macroangiopathic ICA occlusions showed an AUC of 0.77 (*p* < 0.001, CI = 0.65–0.89) (Fig. 3). The highest Youden index was 0.48 at an MTT ratio of 1.36 (sensitivity 48%, specificity 100%). In favor of a higher sensitivity compared to specificity within the clinical context, a cut-off of 0.41 was yielded at a value of 1.15 for the MTT ratio (sensitivity 73%, specificity 68%) based on the results of the Youden index.

Multinomial logistic regression for pretherapeutic parameters showed an overall model performance with χ^2^(10) = 82.078, *p* < 0.001 that indicates an improvement in fit over the null model. Goodness of fit showed a Pearson’s χ^2^(126) = 58.819, *p* = 1.0 and a deviance χ^2^(126) = 49.178, *p* = 1.0, which indicated a good fit of the model. A pseudo-Nagelkerkes R^2^ =0.82 was yielded. There was a significant contribution of age (*p* = 0.006), hypertension (*p* = 0.021) and atrial fibrillation (*p* < 0.001) in this model (Table 5) and 95.7% of embolic occlusions, 90.0% of macroangiopathic occlusions and 50.0% of dissections were correctly predicted by the model.

**Table 5:** Multinominal logistic regression

	−2 Log-Likelihood	χ^2^	*p*-value
MTT ratio	52.84	3.66	0.160
Sex	54.27	5.08	0.079
Hypertension	56.86	7.68	0.021
Age	59.35	10.18	0.006
Atrial fibrillation	83.67	34.49	< 0.001

Overall model with χ^2^(10) = 82.078, *p* < 0.001 and pseudo-Nagelkerkes R^2^ = 0.82

*MTT* mean transit time

## Discussion

The aim of this study was to evaluate the use of CT‑P parameters to determine the underlying etiology in ICA occlusions. Therefore, we compared 69 consecutive patients with an occlusion of the ICA caused by macroangiopathic, embolic or dissection etiology regarding differences in cerebral perfusion measured with CT‑P.

The underlying etiology of an ICA occlusion is an important factor for the acute treatment of the patient and a prognostic factor for patient outcome [3, 5, 16, 17]. Angiographic imaging may be helpful in determination of the underlying etiology of the ICA occlusion, but noninvasive, pretreatment determination is desirable [18]. In this context, it is useful to evaluate CTA, in which the visualization of the occlusion is very accurate, but the filling defect cannot be imaged as sensitively as in DSA, which may complicate inference of the underlying etiology [19]. Besides imaging of the occlusion itself, the age of patients allows a reference to the cause of ICA occlusion: young patients suffer more frequently from dissection and patients with an older age more frequently show macroangiopathic or embolic ICA occlusions, which is in line with the findings in our current study [10]. Furthermore, some preconditions are suggestive for a specific etiology as atrial fibrillation is common in patients with embolic occlusions and dissections are slightly more frequently found in male patients [20]. We were able to observe these coherences in our study sample as well and could show that there are markedly distinguishable age differences between each of the three etiologies evaluated.

As on the one hand angiographic imaging is an invasive procedure and availability is restricted and on the other hand clinical features like age may not distinguish between embolic and macroangiopathic patients, we considered cerebral perfusion as a possible additional tool to differentiate between the varying etiologies. Due to the slow progression of the underlying stenotic disease, perfusion compensation by collateral supply from the contralateral side is typically seen in macroangiopathic occlusions [21]. In contrast, a sudden decrease in perfusion usually occurs in embolic occlusions or dissections. In addition, the progressive rarefaction of cerebral collaterals with age aggravates the reduced perfusion of the affected area, which is particularly relevant in embolic occlusions, which typically affect an older population with usually worse functional outcome [22, 23]. Accordingly, functional outcome in patients with embolic ICA occlusions compared to other etiologies was statistically non-significant worse (Table 1).

**Fig. 4:**
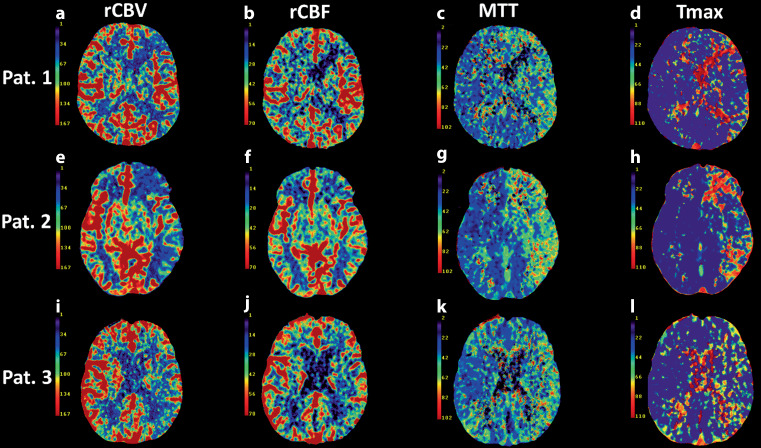
Perfusion parameters of patients with a dissection, an embolic and a macroangiopathic occlusion of the internal carotid artery (ICA). Computed tomography perfusion parameters including *rCBF* (**a**, **e**, **i**), *rCBV* (**b**, **f**, **j**), *MTT* (**c**, **g**, **k**) and *Tmax* (**d**, **h**, **l**) of a patient with a dissection (*Pat.* *1*, **a**–**d**), an embolic (*Pat.* *2*, **e**–**h**) and a patient with a macroangiopathic occlusion (*Pat.* *3*, **i**–**l**) of the left ICA each, the patient with an embolic ICA occlusion had the proportionally most pronounced left hemispheric perfusion deficit. *Pat.* patient, *rCBV* relative cerebral blood volume, *rCBF* relative cerebral blood flow, *MTT* mean transit time, *Tmax* time to maximum

Collateralization may be depicted by CT‑P, therefore, our focus was to evaluate perfusion parameters with respect to relevant differences in the various etiologies of ICA occlusions (Fig. 4). We were able to show that it is possible to distinguish between embolic and macroangiopathic occlusions based on MTT ratio (ratio of mean values of the affected MCA territory divided by the unaffected MCA territory). There was a higher MTT ratio in embolic occlusions, which reflects the average time required to perfuse a certain brain region (MTT = CBV/CBF) and may be caused by less marked collaterals due to the suddenness of occlusion in embolic occlusions compared to macroangiopathic occlusions [24]. This is in line with the assumption of Lin et al. who also concluded a better collateralization in macroangiopathic occlusions compared with embolic occlusions [25]. Because of increased cerebral collateralization in younger patients, differentiation between dissections and embolic occlusions might also be expected, but we could not demonstrate this at present. Chen et al. investigated cerebral perfusion in ICA dissection and concluded that cerebral perfusion is a poor predictor of stroke in ICA dissection, suggesting that the incidence and severity of stroke in dissections is more multifactorial [26].

Finally, in this evaluation, MTT ratio as the only perfusion parameter is shown to be a useful parameter to allow an estimation of the pathogenesis of stroke in ICA occlusions. For this purpose, the establishment of a cut off is useful, which is defined at a value of 1.15 for the MTT ratio (sensitivity 73%, specificity 68%) to distinguish between embolic and macroangiopathic occlusions. Further studies with larger patient collectives should be performed to confirm or adjust the cut off. The model calculated with multinomial regression analysis was also able to achieve a good overall explanation of variance with a high goodness of fit. Atrial fibrillation contributed most to the model due to the association with cardioembolism but also the other parameters made a significant contribution to the model’s quality. Nevertheless, studies with larger numbers of cases as well as additional parameters should elicit an optimal pretherapeutic model for predicting the etiology of vascular occlusion. In this context, approaches using machine learning should also be included.

### Limitations

We have limitations to admit. First, this study is limited by the total number of patients as well as the different group sizes of the individual subgroups. Therefore, a basically reasonable propensity score matching was not possible. Another limitation is that not every patient has undergone angiography, which is considered the most sensitive imaging technique for determining the etiology of the ICA occlusion; however, all patients have undergone vascular imaging, which has been integrated into the multidisciplinary process of determining etiology in accordance with current guidelines, thus allowing for its determination with a reasonable level of certainty. Volumetry of infarction could not be performed because the CT‑P consists of only two adjacent slices. Furthermore, there was no randomization by intention.

## Conclusion

Our data imply that it is possible to differentiate between patients with embolic and macroangiopathic vascular occlusions based on MTT ratio and to define a corresponding cut off. Differentiation from patients with dissection versus the other etiologies was not possible by perfusion parameters in our sample. Finally, a definitive determination of etiology based on a specific cut-off is considered difficult, but MTT ratio should be included in a further elaborated model to allow a reliable determination of the underlying etiology.
